# Characterisation of a highly potent and near pan-neutralising anti-HIV monoclonal antibody expressed in tobacco plants

**DOI:** 10.1186/s12977-021-00560-6

**Published:** 2021-06-28

**Authors:** Catherine M. Moore, Melanie Grandits, Clemens Grünwald-Gruber, Friedrich Altmann, Maria Kotouckova, Audrey Y.-H. Teh, Julian K.-C. Ma

**Affiliations:** 1 Hotung Molecular Immunology Unit, Institute for Infection & Immunity, St George’s University of London, Cranmer Terrace, London, SW17 0RE UK; 2 Department of Chemistry, University of Natural Resources and Applied Life Sciences, Vienna, Austria

**Keywords:** HIV, Monoclonal antibodies, bNAbs, Plants, Molecular pharming, Immunotherapy

## Abstract

**Background:**

HIV remains one of the most important health issues worldwide, with almost 40 million people living with HIV. Although patients develop antibodies against the virus, its high mutation rate allows evasion of immune responses. Some patients, however, produce antibodies that are able to bind to, and neutralise different strains of HIV. One such ‘broadly neutralising’ antibody is ‘N6’. Identified in 2016, N6 can neutralise 98% of HIV-1 isolates with a median IC_50_ of 0.066 µg/mL. This neutralisation breadth makes N6 a very promising therapeutic candidate.

**Results:**

N6 was expressed in a glycoengineered line of *N. benthamiana* plants (pN6) and compared to the mammalian cell-expressed equivalent (mN6). Expression at 49 mg/kg (fresh leaf tissue) was achieved in plants, although extraction and purification are more challenging than for most plant-expressed antibodies. *N*-glycoanalysis demonstrated the absence of xylosylation and a reduction in α(1,3)-fucosylation that are typically found in plant glycoproteins. The N6 light chain contains a potential *N*-glycosylation site, which was modified and displayed more α(1,3)-fucose than the heavy chain. The binding kinetics of pN6 and mN6, measured by surface plasmon resonance, were similar for HIV gp120. pN6 had a tenfold higher affinity for FcγRIIIa, which was reflected in an antibody-dependent cellular cytotoxicity assay, where pN6 induced a more potent response from effector cells than that of mN6. pN6 demonstrated the same potency and breadth of neutralisation as mN6, against a panel of HIV strains.

**Conclusions:**

The successful expression of N6 in tobacco supports the prospect of developing a low-cost, low-tech production platform for a monoclonal antibody cocktail to control HIV in low-to middle income countries.

**Graphic abstract:**

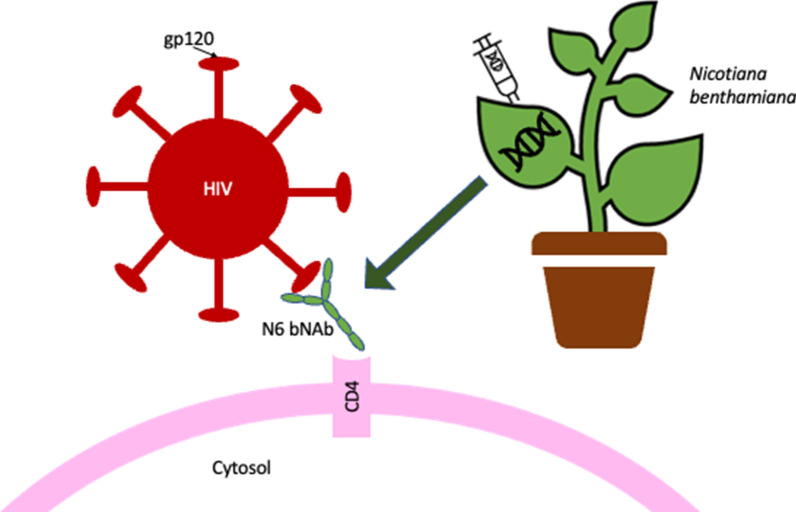

## Background

HIV has been responsible for more than 32 million deaths, and infects an estimated 1.7 million people per year, mainly in Eastern and Southern Africa [1]. Despite enormous effort, there is no cure and no vaccine [2]. The current treatment is anti-retroviral therapy (ART), which must be taken according to a strict regimen, otherwise the patient could relapse [3]. Not only is consistent access therefore essential, the treatment is very expensive (~ $10,000/year) [4]. The global spread of resistance to ART means more treatments are urgently needed [5, 6].

Monoclonal antibodies (mAbs) that neutralise HIV have been of widespread interest for almost 30 years [7–11]. Antibodies’ most obvious advantage over ART is that they can direct antibody-dependent cellular cytotoxicity (ADCC), so that in addition to blocking infection, they can also trigger the immune system to kill infected cells. As well as this, antibodies have a longer half-life than ARTs, which means the treatment can be administered less frequently [12]. mAbs are widely used in other medical areas, such as cancer and chronic disease [13–15] but are yet to make an impact in HIV for several reasons. Importantly, mAbs remain costly using conventional manufacturing technologies. Immunotherapy has been estimated to cost $96,731 per year, compared to $10,000 for ARTs [4, 16]. Another important barrier is the diversity of HIV strains, and the virus’s propensity for mutation and escape, necessitating the use of cocktails of multiple mAbs, which would significantly increase the cost with every additional mAb added.

The discovery of broadly neutralising antibodies (bNAbs) in a small group of patients, so-called ‘elite controllers’ [17, 18], took the prospect of using mAbs against HIV a major step closer. Several anti-HIV bNAbs are currently in clinical trials [19]. Current bNAbs are significantly more potent than the early neutralising mAbs. Examples such as VRC01 and 3BNC117, which mimic CD4-gp120 binding, are able to neutralise 91% and 82% of HIV-1 virus strains respectively [20, 21]. Recently, bNAb ‘N6’ with almost pan-neutralisation was discovered in an elite controller patient [22]. N6 binds to more conserved regions of gp120, and tolerates changes in HIV envelope such as glycans attaching to V5, which is a common mechanism for resistance to other bNAbs [22]. The vast neutralisation breadth of N6 has never been reported in any other bNAb and provides the possibility that far fewer mAbs would need to be combined to make a useful and durable anti-HIV product.

A low-cost production platform would be necessary for this treatment to be feasible, however. One such platform, which is gaining traction, is the use of plant biotechnology to turn plants like tobacco into living bioreactors. This approach is simple, scalable, low-tech, and requires a smaller initial investment than traditional drug production platforms [23–26]. One plant-expressed anti-HIV bNAb, 2G12, successfully completed its phase I clinical trial as of 2015 [27]. Producing N6 in tobacco plants could offer LMICs, many of which already have tobacco-growing expertise, the opportunity to produce their own anti-HIV therapeutic production platform for their whole region, given that the plant production system can quickly produce bulk quantities [28, 29].

In this study, the feasibility of producing bNAb N6 in plants was investigated. A glyco-engineered line of *Nicotiana benthamiana (ΔXF*) [30] was used to overcome potential issues with effector function and blood clearance activity [31]. The purified protein was characterised and compared to the same antibody produced by a conventional mammalian cell expression system, in terms of antigen binding, binding kinetics and breadth of viral neutralisation, as well as glycosylation and FcγRIIIa and ADCC activity. Our results suggest that plants can be developed as a scalable, low-cost production platform for N6. Their use could help offset the prohibitive expense of mAb therapies, and the low upstream costs for plant manufacturing could allow the most affected regions to take ownership of their own treatment development programmes.

## Results

### Expression and yield optimisation

Initial expression of N6 in HEK-293 T cells resulted in a yield of 0.6 mg/ml in crude cellular extract. However, using a standard protein A affinity purification resulted in over 90% loss. This could be mitigated by the addition of 0.1% Tween 20 during either the homogenisation stage, the elution stage, or just before filter-sterilising—an approach that was previously reported to prevent protein aggregation during purification [32]. In our case, this allowed purification of over 25% of the expressed antibody.

**Fig. 1:**
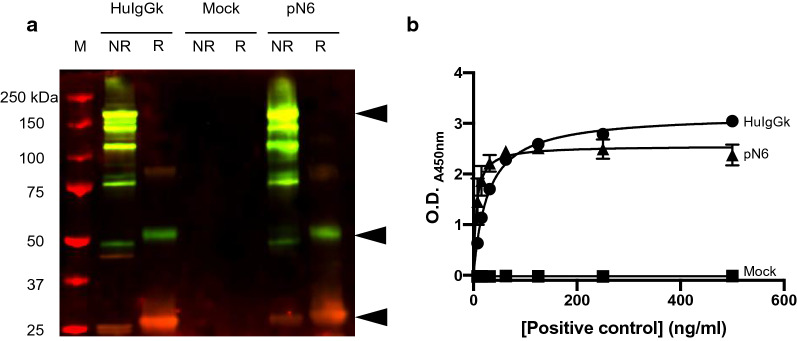
Expression of N6 antibody in *N. benthamiana*. **A** Human IgG kappa (HuIgGk) from human serum (Sigma), mock-infiltrated sample from leaf disk (mock), N6 extracted from leaf disk (pN6), and Precision Plus Protein™ All Blue Pre-stained Protein Standards (M) were run, either reduced (R) or not reduced (NR), on SDS-PAGE before blotting onto nitrocellulose. Heavy chain was detected with mouse anti-human IgG Fc domain and light chain was detected with goat anti-human kappa light chain. Secondary antibodies were donkey anti-mouse with green fluorescent tag, and donkey anti-goat with red fluorescent tag. Black arrows indicate, from top to bottom, fully assembled antibody (yellow), heavy chain (green) and light chain (red). **B** Sandwich ELISA detecting fully assembled antibody in plant crude extract. Leaf disks taken from *N. benthamiana* transiently expressing N6 (triangle), human IgG kappa positive control (circle) or plants mock-infiltrated with infiltration solution only (square) were extracted in PBS and introduced to an ELISA plate coated with goat anti-human IgG Fc domain antibody. Bound antibodies were detected using HRP-conjugated goat anti-human IgG kappa light chain antibody. Representative of 3 biological replicates (i.e. separate plants and infiltration experiments). Each ELISA was performed with 2 technical replicates. Means derived from 2 leaf disks per sample ± S.D. Yields were estimated using Graphpad Prism software, fitting to Michaelis Menton equation

For expression in tobacco, the N6 DNA sequence was codon-optimised (GeneArt, Thermo-fisher) for the *Nicotiana* genus. When the plant-optimised N6 was expressed in tobacco and extracted with 0.1% Tween-20, the yield in crude extract was 49 mg/kg (fresh tissue mass). Figure 1A shows the pN6 antibody in its reduced and non-reduced state on a western blot, detected by anti-human light chain antiserum (red fluorescence) or by anti-human IgG Fc antiserum (green fluorescence). Under non-reducing conditions, the largest band of approximately Mr 180 kDa (top arrow) represents the fully assembled IgG (yellow). A number of smaller bands are also seen, representing either assembly intermediates or degradation fragments. Under reducing conditions, the individual heavy and light chains are detected at approximately Mr 55 kDa and 27 kDa (arrowed) respectively. The positive control is a commercial purified human IgG1κ mAb which gave similar results, and the negative control is the plant extract from a mock-infiltrated plant. The assembly of light and heavy chains was confirmed using a sandwich ELISA, where assembled N6 was detected using anti-heavy chain and anti-light chain antibodies (Fig. 1B).

### HIV gp140 binding kinetics

Preliminary tests to assess binding were performed by a direct ELISA using recombinant, soluble HIV‐1 UG37 gp140 (Centre For AIDS Reagents), as the capture antigen. UG37 gp140 is a variant of gp160 that lacks the transmembrane domain, from the HIV-1 clade A strain 92UG037.

**Fig. 2:**
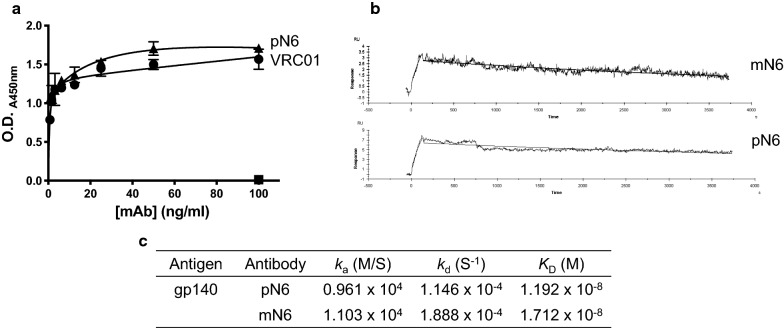
N6 antigen binding assessment. **A** ELISA demonstrating specific binding of anti-HIV antibody to its cognate antigen—gp140. Purified pN6 antibody (triangle), commercially-sourced human IgG1 kappa (square) and VRC01 (circle) were incubated on an ELISA plate coated with gp140. VRC01 (circle) was previously purified from tobacco plants in-house. Bound antibodies were detected using HRP-conjugated goat anti-human IgG Fc domain antibody. Human IgG kappa (square) was included as a negative control at 100 ng/ml only. Representative of 3 biological replicates. ELISAs were performed with 2 technical replicates. Data shown are mean ± S.D. **B** Surface plasmon resonance measuring binding kinetics of pN6 antibody compared to mN6. Protein A was immobilised onto a CM5 chip and N6 antibody was captured to 5000 RU. **C** Calculated association constant (*k*_a_), dissociation constant (*k*_d_) and affinity (*K*_D_) from surface plasmon resonance were estimated using the Langmuir model of binding (1:1), with BIAcore™ Evaluation software. Both versions of N6 bind to gp140 with an equivalent affinity

pN6 binding was titrated from 0.8–100 ng/ml and compared with pVRC01—an anti-HIV broadly neutralising antibody that was previously successfully expressed in *N. benthamiana* [33] (Fig. 2A)*.* Human IgG1κ was included at 100 ng/ml only, as a negative control. The binding of N6 was very similar to that of VRC01, whereas human IgG1κ did not bind at all.

The binding kinetics of pN6 to gp140 was determined using surface plasmon resonance (Biacore). N6 antibodies were bound to a protein A-coated CM5 chip and gp140 was introduced at a flow rate of 40 μL/min. The binding kinetics for mN6 and pN6 *K*_D_s were calculated using the Langmuir 1:1 model of binding (Fig. 2B, C). There was little difference between the association and dissociation constants for mN6 and pN6, consequently the affinities (*K*_D_) were similar—1.712 × 10^–8^ M, and 1.192 × 10^–8^ M respectively.

### Assessment of HIV-neutralisation potency

**Fig. 3:**
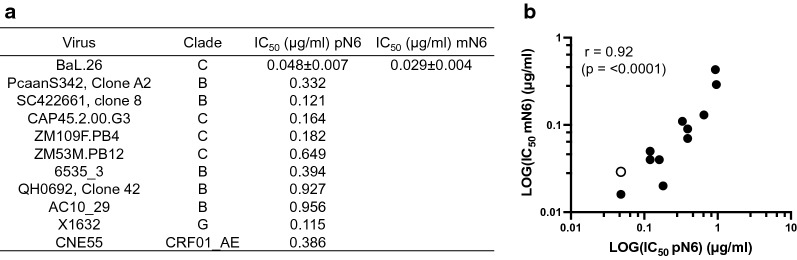
Neutralisation of HIV-1 pseudoviruses by pN6. **A** Neutralisation by pN6 was assessed against a panel of 10 HIV-1 ENV pseudoviruses, and HIV-1 strain BaL.26 as an internal control, using TZM-bl cells. pN6 neutralisation assay was carried out in triplicate, and mN6 BaL.26 set was carried out in duplicate. **B** Correlation of published mN6 IC_50_s with pN6 IC_50_s from pseudovirus neutralisation assay. Open circle denotes assay-derived IC_50_ of pN6 compared with mN6 for BaL.26, rather than published value, as an internal control. Pearson correlation analysis calculated r value of 0.92 (p ≤ 0.0001)

A panel of HIV-1 pseudoviruses were incubated with N6 antibody to assess neutralisation potency, using the TZM-bl assay described in Teh et al. [33]. Neutralisation was determined from transcription of the luciferase reporter gene and IC_50_ was determined (Fig. 3A). HIV BaL.26 was used as a control, and no appreciable difference between the plant- and mammalian cell-derived N6 IC_50_ was observed, both in the ng/ml range. Ten other HIV-1 pseudoviruses were tested, including representatives from clades B and C, and neutralisation by pN6 was observed in each case in the expected range. The results are consistent with those previously reported for N6 expressed in HEK cells [34] and correlate with a r value of 0.92 (p ≤ 0.0001, Pearson correlation) (Fig. 3B).

### Glycosylation analysis

**Fig. 4:**
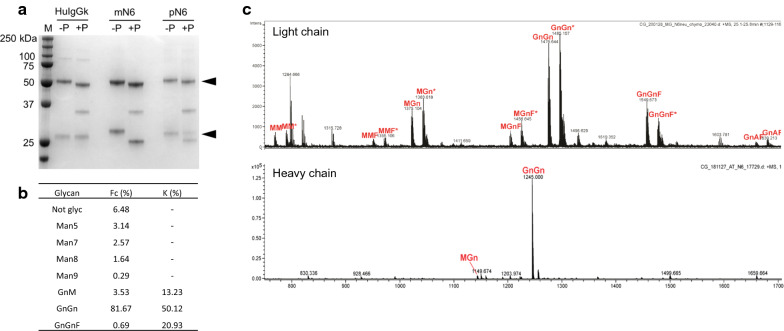
Glycosylation analysis of pN6. **A** PNGaseF assay where 1 µg each of PNGaseF-digested antibody (+P) was compared to undigested antibody (−P), including the positive control HuIgGk (human IgG1 kappa antibody, Sigma). Marker (M) is Precision Plus Protein™ All Blue Pre-stained Protein Standards. Heavy and light chains are indicated by black arrows. PNGase F enzyme visible at 36 kDa. **B** Percent abundance, derived from mass spectrometry, of various glycoforms in the heavy (Fc) and light (K) chains. **C** Mass spectra of pN6 heavy and light chain glycoforms. Purified proteins were analysed by digestion with trypsin followed by LC–ESI–MS. Glycopeptides from the kappa-chain variable region occurred as doubly charged ions, partly with ammonium

A preliminary determination of pN6 and mN6 glycosylation was performed by PNGaseF digestion followed by SDS-polyacrylamide gel electrophoresis (Fig. 4A). This demonstrated a size shift for both the light and heavy chains in both cases, indicating that both the light and heavy chains are *N*-glycosylated. An unrelated mAb (human IgGκ), which was used as a control, had a size-shift in the heavy chain only.

Figure 4B shows the analysis of glycan structure relative abundance on pN6 heavy and light chains, measured by mass spectrometry. The heavy chains (Fig. 4C lower panel) had predominantly mature complex-type glycans (GnGn 81.67%) with a small proportion of high mannose glycosylated heavy chains and 6.48% non-glycosylated. As expected with expression in the *Δ*XF *N. benthamiana* line, no xylosylation was detected and only a small proportion of the heavy chains were fucosylated (0.69%). On the light chain (Fig. 4C upper panel) the majority of the light chains had mature complex-type glycans and there were no high mannose glycoforms. There was no xylosylation, but there was significantly more α(1,3)-fucose glycosylation (20.93%).

### Antibody-dependent cellular cytotoxicity activation

**Fig. 5:**
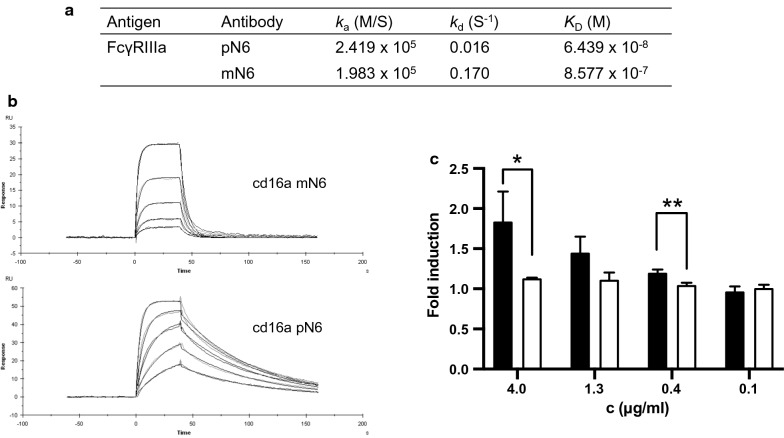
N6 Fc effector function assessment. **A** Binding kinetics of FcγRIIIa to pN6 and mN6 measured by surface plasmon resonance. Protein A was immobilised onto a CM5 chip and N6 antibody was captured to 5000 RU. Association constant (*k*_a_), dissociation constant (*k*_d_) and affinity (*K*_D_) were estimated using the Langmuir model of binding (1:1), with BIAcore™ Evaluation software. **B** Surface plasmon resonance measuring binding kinetics of pN6 compared to mN6. **C** Antibody dependent cellular cytotoxicity (ADCC) assay comparing activation of ADCC by pN6 (black) with mN6 (white). ADCC is detected by reporter effector cells expressing luciferase when activated. Results are from 3 technical replicates ± S.D. Statistical analyses carried out using Graphpad Prism software Student’s T-test. * p ≤ 0.05. ** p ≤ 0.006

The reduction in fucosylation on the heavy chains of pN6 was reflected in the binding kinetics to FcγRIIIa (Fig. 5A, B). In a SPR assay measuring binding of soluble FcγRIIIa to N6 on the solid phase, there was almost one log difference in affinity between pN6 (*K*_D_ 6.439 × 10^–8^ M) and mN6 (*K*_D_ 8.577 × 10^–7^ M). As observed elsewhere, the difference in affinity was largely due to a difference in the dissociation constant *k*_d_ [31].

To investigate if the different binding kinetics of pN6 and mN6 to FcγRIIIa could result in a functional impact, an antibody-dependent cellular cytotoxicity (ADCC) reporter assay was performed, in which activation of effector cells by antibody binding is measured by expression of luciferase (Promega, UK). No activation of effector cells was observed at any of the concentrations of mN6 (p = 0.0633, one-way ANOVA) (Fig. 5C). For pN6 however, there was a significant difference between the concentrations tested (p = 0.0059), at 4.0 and 1.3 µg/ml, the two highest concentrations, there was a significant difference in the induction of effector cell response between pN6 and mN6 (p = 0.0298 and p = 0.0058 respectively).

## Discussion

Current chemotherapy for HIV (a cocktail of antiretroviral drugs), whilst effective, is demanding, with non-adherence contributing towards the emergence of resistance [35–38]. Furthermore, the same anti-retroviral drugs are being used in every HIV intervention, including for treatment, pre- and post-exposure prophylaxis [39]. There are many reasons therefore, why a different class of anti-HIV drug would be welcome.

Whilst mAbs could be used in multiple ways to prevent or treat HIV, there are preferred indications for mAbs. For example, antibodies have been used very effectively to provide immediate, short-term protection against infection, as in passive immunisation against hepatitis A, rapid passive protection in childbirth (against Rhesus antigen) and immediate protection following infectious challenge (rabies post-exposure prophylaxis) [40–42]. mAbs could be used similarly against HIV, replacing or supplementing ART, for example in pre-exposure prophylaxis (PrEP), in the prevention of vertical (mother to child) transmission during childbirth and in post-exposure prophylaxis following accidental exposure to infected body fluid (PEP) [43, 44].

In this report we demonstrated for the first time that the near-pan neutralising anti-HIV antibody N6 can be efficiently expressed in tobacco plants (pN6). The plant-expressed antibodies retain their specificity and affinity for HIV envelope protein, and their neutralisation potency against a panel of HIV pseudoviruses was unaltered. These findings are consistent with other studies that show antibodies produced in tobacco perform as well as those produced in mammalian cell culture [30, 33, 45–47].

pN6 expression in *N. benthamiana* reached 49 mg/kg—an acceptable level for this stage of development where 30 mg/kg is informally regarded as the lower limit for manufacturing feasibility. The extraction of antibody both from plants and HEK cells did, however, require the addition of 0.1% Tween 20, an approach which is reported to help by stopping aggregation, and is commonly added to biopharmaceuticals as a stabiliser [32, 48]. However, addition of a detergent to the extraction buffer is generally undesirable because it adds cost, complicates downstream processing and adds an additional assay to the product specification and analysis. Thus, further optimisation of the extraction and purification process, or of the antibody backbone sequence itself, will probably be required before N6 can be produced commercially in any heterologous expression system.

The only difference between mAbs expressed in plants or mammalian cells lies in the post-translational modification, and most important of these is *N*-glycosylation [49, 50]. None of the plant-derived biopharmaceuticals trialled thus far in humans have demonstrated any immunogenic effect [27, 29, 51–54]. Nevertheless, pN6 was expressed in a glycoengineered *N. benthamiana* line that eliminates xylose and reduces fucose residues on *N*-glycans [30], that are commonly found in plant, but not mammalian glycoproteins. Previous reports have shown that antibodies expressed in plants can show significantly less *N*-glycan heterogeneity than the same antibody expressed in mammalian systems [33, 55] and the same was found for the N6 heavy chain in this study, with 82% displaying the same complex glycoform. Interestingly, N6, in common with some other broadly neutralising anti-HIV antibodies, is glycosylated on the light chain as well as the heavy chain [33]. About 20% of antibodies are *N*-glycosylated in the Fab domain (on heavy and light chains), and while the functional implication of Fab *N*-glycosylation remains unclear, a role in immune modulation [56] and serum half-life [57] has been suggested. pN6 light chain displayed only complex glycoforms, xylosylation was absent, and there was a greater proportion of α(1,3)-fucosylation than found on the heavy chain. These findings are consistent with the kappa chain *N*-glycosylation site being more exposed for post-translational modification than the Fc site.

We and others have shown that IgG mAbs bearing *N*-glycan fucosylation at either the α-1,6 position (in mammals) or the α-1,3 (in plants) have reduced binding affinity to the low affinity FcγRIIIa (CD16a) receptor [31, 58–61] which is found on natural killer cells, neutrophils and monocytes. Expressing an IgG mAb in the *Δ*XF *N. benthamiana* line was shown to restore the interaction with FcγRIIIa receptor [31]. Antibody binding to FcγRIIIa is associated with antibody dependent cellular cytotoxicity (ADCC), an effector mechanism that enhances killing of virally infected cells by these immune cells [60]. pN6 in this study was shown to have approximately 8-times greater affinity to FcγRIIIa than HEK cell expressed version, and this was associated with a significant enhancement of effector cell activation in an ADCC assay. Others have incubated their assay for 18 h to demonstrate ADCC. Here, a 6 h incubation was sufficient [62]. While the role of ADCC for HIV treatment has not been elucidated, engineering bNAbs for enhanced ADCC is valuable, as studies have shown that intact effector functions are often crucial for maximum potency of bNAbs and high ADCC activity is linked to slow disease progression [63–70].

## Conclusions

We have demonstrated that N6, the most broadly neutralising anti-HIV antibody discovered to date, can be efficiently expressed in tobacco plants without any loss of function. pN6 has great potential as a relatively low-cost, yet highly effective, therapeutic for HIV. N6 is the latest HIV bNAb reported to be successfully expressed in plants. VRC01 was among the first bNAbs to be identified [20, 71] and there are two reports of expression in plants [33, 72]. VRC01 has approximately 90% neutralisation coverage in vitro and is being investigated in numerous clinical trials [20, 73]. Thus, evidence for the feasibility of a plant-derived HIV bNAb combination product is mounting and the prospect of developing a low-cost, low-tech production platform for a monoclonal antibody cocktail to control HIV in low- to middle income countries is emerging. The almost pan-neutralising breadth of N6 would reduce the number of other antibodies required in a therapeutic cocktail, which will contribute significantly to keeping costs to a minimum, and has other potential applications such as complementing ART, and prevention of mother-to-child HIV transmission [74].

## Methods

### Cloning

N6 heavy and light chain coding regions (accession numbers KX595109 and KX595112 respectively) were provided by Centre For AIDS Research (CFAR) in vector pCMVR for mN6 expression. N6 heavy and light chain sequences were plant codon-optimised and synthesised by GeneArt (Thermofisher). Restriction sites were removed from the sequence during optimisation. These heavy and light chain sequences were inserted into the pTRAk.6 vector [75] using a cloning system developed in-house. Briefly, N6 heavy and light chain genes were digested with NcoI/XbaI and ligated into pWhite and pBlue entry vectors respectively, and then both inserted, using golden gate cloning (BsaI/BsmBI), into the pTRAk.6 *Agrobacterium* binary vector [75]. The pTRAk.6 vectors were used to transform *Agrobacterium tumefaciens* strain GV3101:pM90RK by electroporation [76].

### *N. benthamiana* infiltration

Plants were germinated and maintained in the greenhouse with a 16/8-h day/night cycle at 24–28 °C and infiltrated after 4–6 weeks of growth. Infiltrations were carried out as described in Teh et al. [33]. Briefly, recombinant *Agrobacterium tumefaciens* were grown until an O.D._600 nm_ of 2–4 was achieved. The bacteria were pelleted and resuspended in infiltration solution (10 mM MgCl_2_, 10 mM MES) at an O.D._600 nm_ of 0.1 and incubated at room temperature for a minimum of 30 min with 200 µM acetosyringone. Plants were infiltrated manually using a syringe or by vacuum infiltration for larger scale expression. Plants were harvested five days post infiltration.

### HEK-293T cell culture

HEK-293T cells were grown in DMEM medium [DMEM High Glucose, +sodium pyruvate (110 mg/L), supplemented with l-glutamine (200 mM), foetal bovine serum 10% and streptomycin (10,000 μg/mL)] at 5% CO_2,_ 37 °C. Cells were transiently transfected with 2 µg (total) of N6 heavy and light chain vectors using FuGENE-HD transfection kit, following manufacturer’s instructions (Promega, cat. #E2311). Supernatants were harvested and filter-sterilised after 72 h to be used immediately or stored at + 4 °C for no more than seven days.

### Western blots

All SDS-PAGE gels and western blots were performed following the Invitrogen NuPAGE manufacturer’s instructions (NuPAGE). 4–12% Bis–Tris SDS-PAGE gels were run in MOPS buffer, blotted onto nitrocellulose and blocked with LI-COR® Odyssey® PBS blocking buffer. Primary antibodies were diluted 1/1000 in Odyssey® PBS blocking buffer from a 1 mg/ml stock. Secondary antibodies were Odyssey® donkey anti-mouse, anti-rabbit, or anti-goat antisera which were tagged with fluorophores 800CW or 680RD, and diluted 1/10,000. Blots were visualised using the LI-COR® Odyssey® CLx scanner and analysed using Image Studio.

### ELISAs

ELISAs were performed as previously described [77]. Briefly, ELISA plates were coated with anti-human IgG1 Fc antiserum (The Binding Site, cat. #AU004) or UG37 gp140 (5 µg/ml in PBS) (CFAR, USA) and blocked with PBS + 5% skimmed milk powder. N6 antibody was titrated two-fold, along with a positive control (500 ng/ml) in PBS + 5% skimmed milk powder and incubated for a minimum of two hours at 37 °C. Primary antibody was anti-human IgG1 light chain kappa antiserum conjugated with HRP (The Binding Site, cat. #AP015), diluted in PBS + 5% skimmed milk powder. Developing solution (3,3 5,5-Tetramethylbenzidine (TMB) Liquid Substrate, Sigma, cat. #T0440) was added and briefly incubated until colour development was complete before stopping with 2 N H_2_SO_4_. Plates were read on the Tecan Infinite F200 Pro. Data were analysed and concentrations calculated with Graphpad Prism 7 using the Michaelis Menton equation for line fitting.

### Antibody purification

N6 antibody was purified as described previously [77]. Briefly, infiltrated plants were homogenised in a blender and filtered through miracloth (Sigma) to remove plant debris. The filtrate was centrifuged for 40 min at 16,000*g*, before filter-sterilising the supernatant through a 0.22 µm filter. Filtrate was purified using affinity chromatography on a Protein A column (Protein A agarose, Sigma, cat. #P2545). Eluates were dialysed overnight at 4 °C and concentrated by buffer exchange in 100 k Centricon® centrifugal filters. Antibodies were filter-sterilised and quantified using Nanodrop™ 2000 spectrophotometer (Thermofisher), before storing at 4 °C, or aliquoting and storing at -80 °C.

### Glycoanalysis

1 µg antibodies were digested with PNGaseF following manufacturer’s instructions (NEB, cat. #P0704). Samples were reduced with 5% β-mercaptoethanol before performing SDS-PAGE. Proteins were identified using InstantBlue™ Coomassie stain (Expedeon, cat. #ab119211). PNGaseF assays were performed in triplicate.

For mass spectrometry, pN6 antibody was trypsin-digested and analyzed by liquid chromatography–electrospray ionization–mass spectrometry as described in Teh et al. [33]. Briefly, samples were resuspended in 80 mM ammonium formiate buffer and run on a BioBasic C18 column with a 5% to 40% 80%-acetonitrile for 45 min, followed by a 15 min gradient from 40 to 90% 80%-acetonitrile, that facilitates elution of large peptides, at a flow rate of 6 µL/min. Peptide identification was performed with maXis 4G ETD (Bruker, Germany) in positive ion mode. Manual glycopeptide searches were made using DataAnalysis 4.0 (Bruker).

### Binding kinetics

Surface plasmon resonance (SPR) was employed to calculate binding kinetics, according to Stelter et al. [31], using the BIAcore™ X-100 instrument (GE healthcare, Chalfont St. Giles, UK). All proteins were diluted/resuspended in HBS-EP + buffer (10 mM HEPES, pH 7.4, 150 mM NaCl, 3 mM EDTA, and 0.05% surfactant P-20) at 25 °C. Protein A (Sigma, cat. #P6031) was immobilised onto a CM5 chip with standard amine coupling, to 5000 response units. Recombinant HIV gp140 was flowed over the chip at a concentration of 80 μg/ml with a flow rate of 40 μl/min for 135 s, followed by 1 h of dissociation time and a regeneration step with 10 mM glycine–HCl (pH 1.5). FcγRIIIa (R&D Systems, USA) was applied in multiple concentrations (1, 0.5, 0.25, 0.125 and 0.0625 μM) 40 s at a flow rate of 50 µl/min, followed by 120 s of dissociation and a regeneration step with 10 mM glycine–HCl (pH 1.5). All referenced and blanked sensograms were fitted to Langmuir model of binding (1:1), using BIAcore™ Evaluation software.

### HIV neutralisation assays

TZM-bl assays were adapted from Wei et al., 2003 and Montefiori, 2005 [78, 79]. HIV-1 pseudovirus stocks were generated by transfecting HEK-293T cells. For neutralization assays bNAbs were diluted to 20 µg/mL and a three-fold serial dilution in triplicates was performed in flat-bottom 96 well plates. Pseudovirus at a dilution translating into 20 × RLU of the background control were added to each well, except the cells-only control. After 1 h incubation, 10^4^ TZM-bl cells, containing DEAE dextran, were added to each well and plates were incubated (37 °C, 5% CO_2_). After 48 h the supernatant was removed, and cells were washed with PBS prior to adding lysis buffer (Promega, cat. #A8261). The plate was kept at − 80 °C overnight to ensure complete virus inactivation. After thawing, the cell lysate was mixed 1:1 with Bright-Glo luciferase substrate (Promega Luciferase Assay System, cat. #E2610) in a black flat bottom 96 well plate. Luminescence was measured using a GloMax plate reader (96 Microplate Luminometer, Promega, USA). IC_50_s were compared to published data available in the CATNAP database [34].

### Antibody-dependent cellular cytotoxicity assays

To determine the ability of the bNAbs to activate ADCC, Promega’s ADCC Reporter Assay for the V-variant was used, which included effector cells (Jurkat cell line stably expressing human FcγRIIIa V158 and NFAT-induced luciferase—cat. #G7015). A three-fold dilution row of each respective bNAb was performed in sterile white flat bottom 96 well plates with a 4 μg/ml starting concentration. A no-antibody control and substrate-only control was included on each plate. An equal volume of recombinant protein gp140 was added to each well and plates were incubated for 1 h at 37 °C (5% CO_2_). ADCC effector cells were thawed, added to ADCC assay medium, and an equal volume of cells was added to each well. Plates were incubated for 6 h, then left at room temperature for 20 min before adding Bio-Glo Luciferase Substrate (Promega, cat. #E2610). After 5 min luminescence was measured using a GloMax-Multi Detection System (Promega, USA). Biological triplicates of the assay were performed. To calculate fold induction the following equation was used:

$$Fold\;induction = \frac{{RLU\left( {induced - substrate~only} \right)}}{{RLU\left( {no\;antibody\;control - substrate\;only} \right)}}$$

## Data Availability

Data and materials provided in manuscript.

## Abbreviations

ELISA: Enzyme-Linked Immunosorbent Assay
PBS: Phosphate-buffered saline
O.D.: Optical density
ADCC: Antibody-dependent cellular cytotoxicity
SPR: Surface plasmon resonance
pN6: Plant-expressed N6
mN6: Mammalian cell-expressed N6
HIV: Human immunodeficiency virus
AIDS: Auto-immune deficiency syndrome
ART: Anti-retroviral therapy
bNAbs: Broadly neutralising antibodies
mAb: Monoclonal antibody
RU: Response units
RLU: Relative light units
HRP: Horse radish peroxidase
SDS PAGE: Sodium dodecyl sulphate poly-acrylamide gel electrophoresis
DMEM: Dulbecco’s Modified Eagle’s Medium
RNAi: RNA interference
HEK-293T: Human Embryonic Kidney
IgG: Immunoglobulin G
GlcNac/Gn: *N*-Acetylglucosamine
Mann/M: Mannose
XF: β(1,2)-Xylose, α(1,3)-fucose
ANOVA: Analysis of variance
